# Anti-proliferative activity of the quassinoid NBT-272 in childhood medulloblastoma cells

**DOI:** 10.1186/1471-2407-7-19

**Published:** 2007-01-25

**Authors:** André O von Bueren, Tarek Shalaby, Julia Rajtarova, Duncan Stearns, Charles G Eberhart, Lawrence Helson, Alexandre Arcaro, Michael A Grotzer

**Affiliations:** 1 Neuro-Oncology Program, University Children's Hospital, Zurich, Switzerland; 2 Department of Pathology, Johns Hopkins University, Baltimore, Maryland, USA; 3 TAPESTRY Pharmaceuticals, Boulder, Colorado, USA; 4 Division of Clinical Chemistry and Biochemistry, University Children's Hospital, Zurich, Switzerland

## Abstract

**Background:**

With current treatment strategies, nearly half of all medulloblastoma (MB) patients die from progressive tumors. Accordingly, the identification of novel therapeutic strategies remains a major goal. Deregulation of c-MYC is evident in numerous human cancers. In MB, over-expression of c-MYC has been shown to correlate with anaplasia and unfavorable prognosis. In neuroblastoma – an embryonal tumor with biological similarities to MB – the quassinoid NBT-272 has been demonstrated to inhibit cellular proliferation and to down-regulate c-MYC protein expression.

**Methods:**

To study MB cell responses to NBT-272 and their dependence on the level of c-MYC expression, DAOY (wild-type, empty vector transfected or c-MYC transfected), D341 (c-MYC amplification) and D425 (c-MYC amplification) human MB cells were used. The cells were treated with different concentrations of NBT-272 and the impact on cell proliferation, apoptosis and c-MYC expression was analyzed.

**Results:**

NBT-272 treatment resulted in a dose-dependent inhibition of cellular proliferation (IC50 in the range of 1.7 – 9.6 ng/ml) and in a dose-dependent increase in apoptotic cell death in all human MB cell lines tested. Treatment with NBT-272 resulted in up to 90% down-regulation of c-MYC protein, as demonstrated by Western blot analysis, and in a significant inhibition of c-MYC binding activity. Anti-proliferative effects were slightly more prominent in D341 and D425 human MB cells with c-MYC amplification and slightly more pronounced in c-MYC over-expressing DAOY cells compared to DAOY wild-type cells. Moreover, treatment of synchronized cells by NBT-272 induced a marked cell arrest at the G1/S boundary.

**Conclusion:**

In human MB cells, NBT-272 treatment inhibits cellular proliferation at nanomolar concentrations, blocks cell cycle progression, induces apoptosis, and down-regulates the expression of the oncogene c-MYC. Thus, NBT-272 may represent a novel drug candidate to inhibit proliferation of human MB cells *in vivo*.

## Background

Medulloblastomas (MB) are the most common malignant brain tumors in children and constitute 20% of all pediatric brain tumors [1]. With current treatment strategies, nearly half of all patients die from progressive tumors. Accordingly, the identification of novel therapeutic strategies remains a major goal.

The c-MYC oncoprotein plays a pivotal role as a regulator of tumorigenesis in numerous human cancers of diverse origin [2-5]. In childhood MB, c-MYC gene amplification has been demonstrated in ~8% of primary tumors [6-11]. Disparity between c-MYC gene copy number and c-MYC mRNA expression level in primary MB tumors and MB cell lines indicates the presence of alternative mechanisms to gene amplification in up-regulating c-MYC expression [12,13]. High c-MYC mRNA expression and c-MYC gene amplification have been suggested to be indicators of poor prognosis in MB [6,9,11-18]. Furthermore, high c-MYC mRNA expression was demonstrated to be significantly associated with tumor anaplasia [19,20].

Quassinoid analogues, such as bruceantin, are capable of inducing an array of biological responses [21,22], including inhibition of protein synthesis [23]. Such an inhibition has been shown to occur via interference at the peptidyltransferase site, thus preventing peptide bond formation [24]. It has been shown in two independent studies that bruceantin is able to down-regulate c-MYC protein expression in a panel of leukemia, lymphoma, and myeloma cell lines [25,26]. Cell lines expressing high levels of c-MYC oncoprotein were most sensitive to bruceantin-mediated effects [25]. Bruceantin has been evaluated in three separate phase I clinical trials with various types of solid tumors [27-29]. Side effects were relatively few and included hypotension, nausea, vomiting, and moderate hematological toxicity. However, in two phase II clinical trials bruceantin failed to prove effective in metastatic breast carcinoma [30] and in advanced malignant melanoma [31].

Based on the studies with bruceantin, proprietary quassinoid analogues have been designed and their *in vitro *cytotoxic activities have been compared with bruceantin by using the MTT assay in a panel of cell lines. The lipophilic small molecule NBT-272 was found to be 2–10 fold more potent than bruceantin in a variety of cancer cell lines [32]. In neuroblastoma – an embryonal tumor with biological similarities to MB – the quassinoid NBT-272 has been demonstrated not only to inhibit cellular proliferation but also to down-regulate c-MYC protein expression [32]. In the current study, we examined the effects of NBT-272 in human MB cell lines expressing different levels of c-MYC.

## Methods

### Human MB cell lines

DAOY (wild-type), DAOY V11 (empty vector transfected) and DAOY M2 (c-MYC vector transfected) human MB cells have been described previously [20]. D341 and D425 human MB cells were the kind gift of Dr Henry Friedman, Duke University, Durham, NC, USA. All MB cells were cultured in Richter's zinc option medium/10% fetal bovine serum (non-essential amino acids were added to the medium of D341 and D425 cells to a final concentration of 1%, and G418 was added to the medium of DAOY V11 and DAOY M2 to a concentration of 500 μg/ml). All cell cultures were maintained at 37°C in a humidified atmosphere with 5% CO_2_.

### Real-time quantitative polymerase chain reaction

10^6 ^cells growing in their mid-log phase were treated with NBT-272 at concentrations indicated and harvested after 24 h. Total RNA isolation, reverse transcription reactions, and RT-PCR were performed as described previously [12,33]. Kinetic real-time PCR quantification of c-MYC mRNA was performed using the ABI Prism 7700 Sequence Detection System (Applied Biosystems, Rotkreuz, Switzerland), as described previously [34]. Primers and probes for c-MYC and the endogenous control 18S rRNA were purchased from Applied Biosystems (Rotkreuz, Switzerland). For each PCR run, a master mix was prepared containing 200 nM of each primer, 400 nM probe, and 500 ng cDNA in a final volume of 25 μl. The thermal cycling conditions comprised an initial denaturation step at 95°C for 10 min, and 50 cycles at 95°C for 15 s and 60°C for 1 min. Experiments were performed in triplicate for each data point. Relative expression of c-MYC mRNA was calculated by using the comparative C_T _method [35].

### Western blot analysis

The expression of c-MYC protein was assessed by Western blot analysis. Briefly, 10^6 ^cells growing in their mid-log phase were treated with NBT-272 at concentrations indicated and harvested after 24 h and whole-cell pellets were lysed with lysis buffer (1 ml/10^7 ^cells, 50 mM Tris-HCl buffer [pH 8.0], 150 mM NaCl, 1% (w/v) Nonidet P40, 0.1% (w/v) sodium deoxycholate, 0.1% (v/w) sodium dodecylsulfate, 1 mM EDTA, and 1 mM EGTA) containing protease inhibitors (Complete, Roche; Basel, Switzerland) and incubated on ice for 30 min. After measuring the protein concentration by the BCA method (Pierce; Rockford, USA), 12 μg total protein lysate was separated by 10 % SDS polyacrylamide gels and the gels were subjected to immunoblotting. Nonspecific binding sites were blocked by 3 h incubation in TBST (10 mM Tris pH 8.0, 150 mM NaCl, 0.05% (w/v) Tween 20) supplemented with 5% (w/v) nonfat milk powder. Membranes were incubated overnight at 4°C with a 1:2000 dilution of c-MYC polyclonal primary antibody (Santa Cruz Biotechnology; Heidelberg, Germany). Membranes were then washed three times at room temperature in TBST for 30 min each time, and bound Ig was detected using anti-isotype monoclonal secondary antibody coupled to horseradish peroxidase (Santa Cruz Biotechnology; Heidelberg, Germany). The signal was visualized by enhanced chemiluminescence ECL (Amersham Biosciences; Dübendorf, Switzerland) and autoradiography. Then, immunoblotting with a 1:5000 dilution of a monoclonal primary β-actin antibody (Sigma; Basel, Switzerland) was performed, to verify equivalent amounts of loaded protein. For densitometry, the zymographic profiles of the gels were scanned. Relative band intensities were determined using Quantity One analysis software (Bio-Rad).

### c-MYC transcription factor binding activation assay

Nuclear protein extracts were obtained from human MB cells by using the BD™ TransFactor Extraction Kit (BD Clontech, Basel, Switzerland) according to the manufacturer's instructions. Aliquots of nuclear protein extracts were stored at -80°C. The activation of c-MYC was measured by using the Mercury TransFactor assay (BD Clontech, Basel, Switzerland), an enzyme-linked immunosorbent assay (ELISA)-based assay [36], according to the manufacturer's instructions. Briefly, 5 μg of nuclear protein samples were incubated for 1 h in a 96-well plate coated with an oligonucleotide which codes for the c-MYC consensus binding site sequence and to which c-MYC contained in nuclear extracts specifically binds. After washing, antibody directed against c-MYC DNA complex (1:1000 dilution) was added to these wells and incubated for 1 h. Following incubation for 1 h with a secondary horseradish peroxidase-conjugated antibody (1:1000 dilution), specific binding was detected by colorimetric estimation at 450 nm using either mutant DNA or no protein addition controlled for nonspecific binding.

### Cytotoxicity assay

Exponentially growing human MB cells (5 × 10^3 ^cells/well) were cultured in 96-well plates in the presence of different concentrations of NBT-272 for 24 h. Untreated cells were used as controls. A colorimetric 3-(4,5-dimethylthiazol-2-yl)-5-(3-carboxymethoxyphenyl)-2-(4-sulfophenyl)-2H-tetrazolium inner salt (MTS) assay (Promega; Wallisellen, Switzerland) was used to quantitate cell viability as previously described [37]. Briefly, 100 μl of target cell suspension was added to each well of 96-well microtiter plates, and each plate was incubated at 37°C in a humidified 5% CO_2 _atmosphere. Following incubation, 10 μl of MTS working solution was added to each culture well, and the cultures were incubated for 4 h at 37°C in a humidified 5% CO_2 _atmosphere. Each experiment was performed in triplicate. The absorbance values of each well were measured with a microplate spectrophotometer (Molecular Devices; Sunnyvale, CA, USA) at 490 nm. IC50 values were calculated by using the GraphPad Prism software.

### Apoptosis assay

Exponentially growing human MB cells were exposed to different concentrations of NBT-272 for 24 h. Untreated cells were used as controls. A photometric enzyme-immunoassay (Cell Death Detection ELISA; Roche Diagnostics, Basel, Switzerland) was used for the quantitative determination of cytoplasmic histone-associated DNA fragments, as described previously [37]. In brief, cell lysates of control and NBT-272-treated cells were placed in a StreptAvidin-coated microtiter plate. A mixture of biotin-labeled monoclonal histone antibody and peroxidase-conjugated monoclonal DNA antibody was then added, followed by incubation for 2 h. After washing to remove unbound antibodies, the amount of nucleosomes was quantified photometrically. The cell death nucleosomes enrichment factor was calculated by dividing the absorbance of treated cells by the absorbance of untreated control cells.

### Cell cycle analysis

The percentages of cells in the different phases of the cell cycle were determined by evaluating DNA content according to methods described [38]. To arrest cells at the G1/S border, DAOY, D341, and D425 MB cells were synchronized in a medium containing 2 mM hydroxyurea (Sigma) for 14 h as described previously [39]. Cells were then transferred into fresh, hydroxyurea-free medium, or medium containing 0.4 μg/ml NBT-272. Control untreated or NBT-272 treated cells were harvested 0, 8, 16, and 24 h after release from hydroxyurea. After washing twice in PBS 1×, the cells were stained with a solution containing 50 μg/ml propidium iodide (Becton-Dickinson; Allschwil, Switzerland) and 100 U/ml RNase A (Qiagen; Hombrechtikon, Switzerland) in PBS 1× for 30 min at room temperature. A total of 30'000 events per sample were acquired. Flow cytometric analysis was performed on a FACSCalibur flow cytometer (BD Biosciences; Allschwil, Switzerland) with CELLQuest software (BD Biosciences). The percentages of the cells in the different phases of the cell cycle were calculated on linear PI histograms using the mathematical software ModFit LT 2.0 (Verity Software House; Topsham, ME, USA).

### Statistical analysis

All data are expressed as mean ± SD. Student's t-test and one-way ANOVA were used to test statistical significance. *P *< 0.05 was considered to be significant. GraphPad Prism 4 software (San Diego, CA, USA) was used to calculate IC50 concentrations and to compare the fitted midpoints (log IC50) statistically.

## Results

### c-MYC expression in human MB cell lines

**Figure 1:**
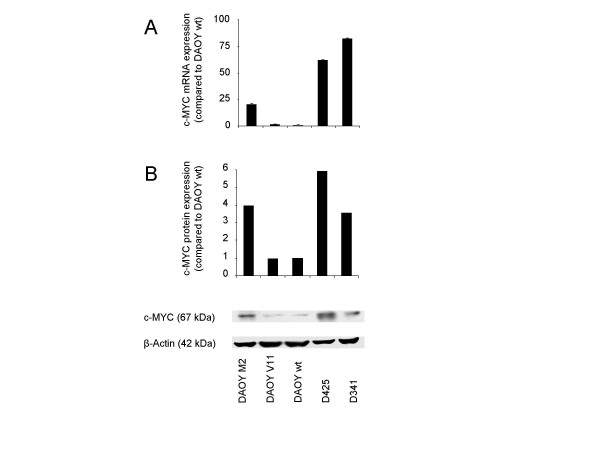
**c-MYC mRNA (A) and protein (B) expression of the MB cell lines determined by quantitative RT-PCR and Western blot analysis**. Values represent the fold-increase of c-MYC mRNA (n = 3; ± SD) and c-MYC protein expression (representative of two to three independent experiments) relative to the DAOY wt cells.

To test the effects of NBT-272 on human MB cells, we selected three human MB cell lines with different levels of c-MYC expression [12,13]. DAOY human MB cells harbor a single copy of c-MYC and display low c-MYC mRNA and protein expression. In contrast, D341 and D425 human MB cells are c-MYC amplified and express high c-MYC mRNA and protein. To study a potential influence of c-MYC expression on cell responses to NBT-272 more specifically, we included DAOY human MB cells transfected to express different levels of c-MYC (DAOY wild-type, DAOY transfected with empty vector, and DAOY transfected with c-MYC) [20]. By using RT-PCR analysis, the c-MYC mRNA expression level was found to be ~20-fold higher in DAOY M2 compared to DAOY wt, or DAOY V11 (Figure 1A). D341 and D425 displayed even higher c-MYC mRNA expression levels (Figure 1A). Using Western blot analysis, c-MYC protein expression was found to be 4-fold higher in DAOY M2 compared to DAOY wt, or DAOY V11. D341 had similar c-MYC protein expression to DAOY M2, whereas D425 had higher c-MYC protein expression (Figure 1B).

### NBT-272 treatment results in dose-dependent suppression of MB cell proliferation

**Figure 2:**
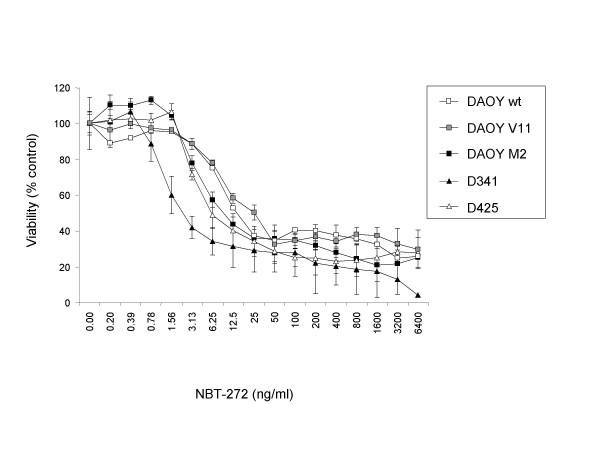
**NBT-272 mediated inhibition of cellular proliferation as determined by the MTS assay**. Cells were incubated for 24 h with various concentrations of NBT-272 or with solvent only. Values represent the mean percentage of viability (representative from two independent experiments) compared with solvent only treated cells ± SD (n = 3; ± SD).

To test whether treatment with NBT-272 alters proliferation of MB cells, we incubated the five human MB cell lines (DAOY wt, DAOY V11, DAOY M2, D341, and D425) with various concentrations of NBT-272 for 24 h and assessed cell viability by using the MTS assay (Figure 2). Treatment with NBT-272 resulted in a dose-dependent cytotoxic response in all MB cell lines tested with an IC50 at NBT-272 concentration of 1.7 – 9.6 ng/ml (D341, 1.7 ng/ml; D425, 4.3 ng/ml; DAOY wt, 8.7 ng/ml; DAOY V11, 9.6 ng/ml; DAOY M2, 4.7 ng/ml) (Figure 2). All MB cells were sensitive to NBT-272 treatment, and their IC50 values were in the low nanomolar range. c-MYC over-expressing DAOY M2 cells, were slightly more sensitive to NBT-272 treatment compared to DAOY wt or DAOY V11 respectively (DAOY M2, IC50: 4.7 ng/ml vs. DAOY V11, IC50: 9.6 ng/ml, p = 0.15). However, these differences were statistically not significant.

### NBT-272 treatment of human MB cells results in down-regulation of c-MYC protein expression

**Figure 3:**
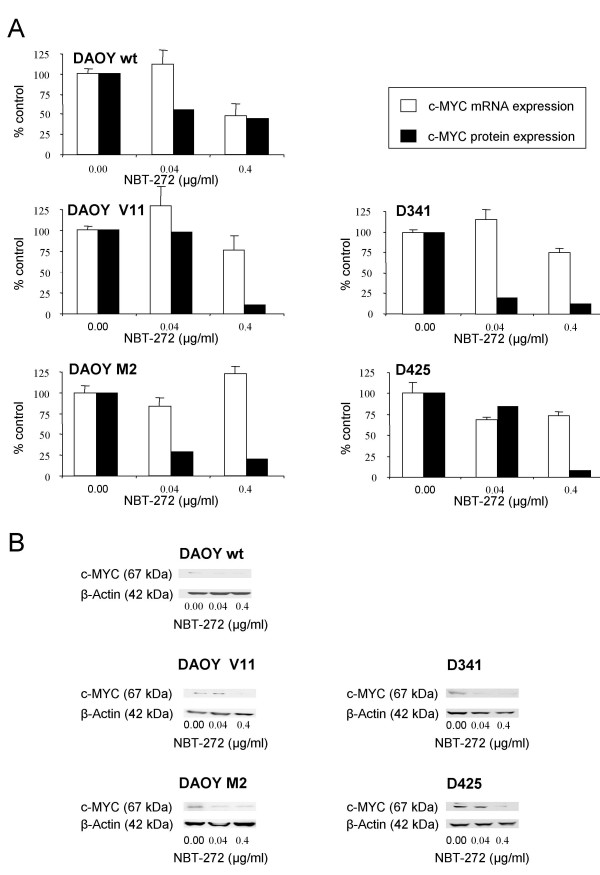
**NBT-272 treatment of human MB cells results in down-regulation of c-MYC protein**. NBT-272 treatment (24 h) of MB cells resulted in no consistent changes of c-MYC mRNA expression as determined by quantitative RT-PCR (A), but in a decrease of c-MYC protein expression as determined by Western blot analysis (A, B). Values represent the percentage of c-MYC mRNA (n = 3; ± SD) and c-MYC protein expression (representative from two independent experiments) compared with solvent only treated cells.

We then determined whether NBT-272 treatment down-regulates c-MYC expression. NBT-272 (0.04 or 0.4 μg/ml) treatment for 24 h resulted in no consistent down-regulation of c-MYC mRNA expression as determined by real time RT-PCR (Figure 3A). However, at the protein level, the effects of NBT-272 were clearly detectable. NBT-272 (0.4 μg/ml) treatment for 24 h resulted in a decrease in c-MYC protein level to 47% in DAOY wt, 11% in DAOY V11, 21% in DAOY M2, 13% in D341, and 9% in D425 (Figure 3A, B). These results indicate that NBT-272 has only minor effects on the c-MYC mRNA levels, but mainly acts at the level of c-MYC protein expression.

### NBT-272 treatment results in a reduction of c-MYC binding activity

**Figure 4:**
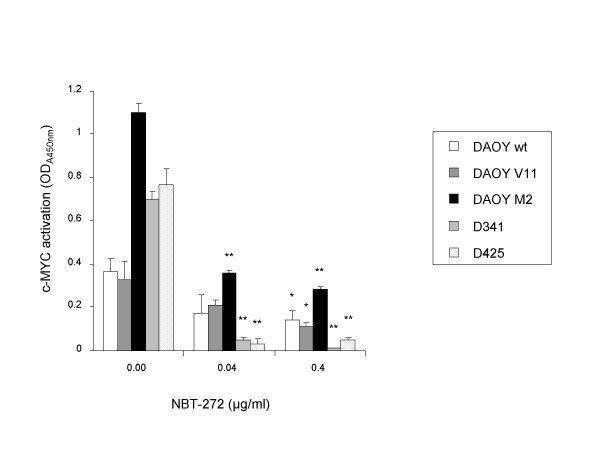
**NBT-272 treatment of human MB cells results in reduced c-MYC binding activity**. NBT-272 treatment (24 h) of MB cells resulted in a reduction of c-MYC binding activity as determined by the ELISA-based TransAM-c-MYC activity assay. Data show the mean absorbance ± SD (n = 3; ± SD). *Significantly different from controls (solvent only treated cells), determined by one-way ANOVA (**:P < 0.005, *:P < 0.05).

To examine whether treatment with NBT-272 alters c-MYC binding activity, we incubated the five human MB cell lines with NBT-272 at various concentrations (0, 0.04, and 0.4 μg/ml) for 24 h and measured binding activity. NBT-272 (0.4 μg/ml) treatment for 24 h reduced c-MYC binding activity significantly in all MB cells tested (Figure 4).

### Induction of apoptosis by NBT-272 in MB cells

**Figure 5:**
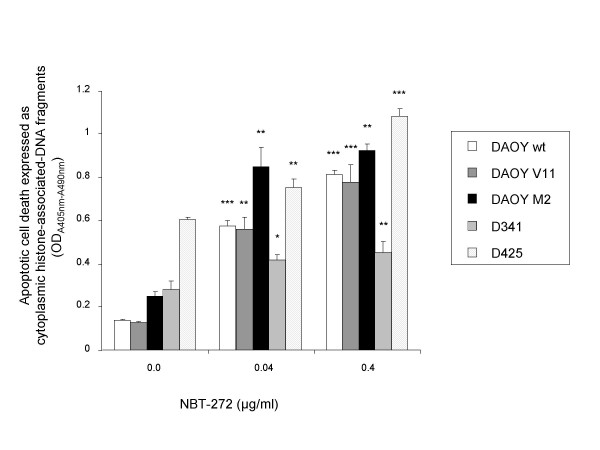
**NBT-272 treatment (24 h) of human MB cells results in an increase of apoptotic cell death**. Values represent the mean absorbance of cytoplasmatic histone-associated-DNA fragments (representative from two independent experiments) compared with solvent only treated cells ± SD (n = 3; ± SD). *Significantly different from control values, determined by one-way ANOVA (***:P < 0.001, **:P < 0.005, *:P < 0.05).

The induction of apoptotic cell death upon treatment with NBT-272 was next assessed. In all MB cell lines tested, NBT-272 treatment (0.04 and 0.4 μg/ml for 24 h) induced apoptosis (Figure 5). Compared with untreated controls, the NBT-272 mediated increase in apoptosis was significant in DAOY wt (425% and 603%), DAOY V11 (443% and 613%), DAOY M2 (343% and 375%), D341 (147% and 162%), and in D425 human MB cells (124% and 178%). DAOY M2 cells were characterized by a higher basal apoptotic activity, when compared to DAOY wt and DAOY V11 cells. Interestingly, the apoptosis-inducing effects of NBT-272 were most prominent in MB cells displaying low c-MYC expression (DAOY wt, DAOY V11). In summary, apoptotic cell death does not appear to contribute equally to the reduction in viability in the MB cell lines under study.

### Effects of NBT-272 on the cell cycle in MB cells

**Figure 6:**
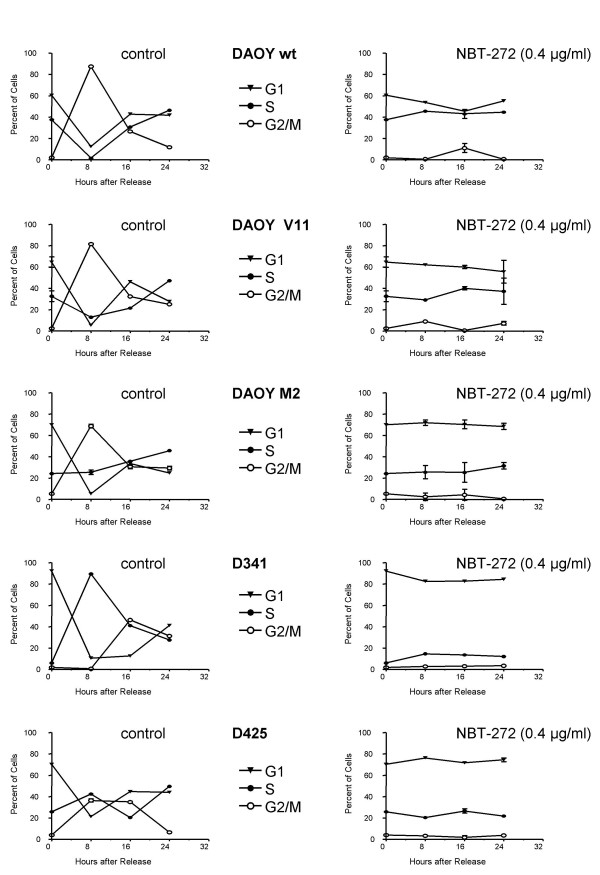
**Block of the cell cycle progression in synchronized MB treated with NBT-272**. MB cells were synchronized by hydroxyurea treatment as described in Methods and treated with NBT-272 or the same volume of medium as control. FACS analysis of NBT-272 and control cells carried out 0, 8, 16, and 24 h posttreatment. Results are presented as the mean percentages of cells in G1, S, and G2/M ± SD (n = 2; ± SD).

To determine possible effects of NBT-272 on cell cycle regulation, the cellular DNA content was assessed using flow cytometry. Under conditions where induction of apoptosis was observed (Figure 5), NBT-272 treatment (0.04 and 0.4 μg/ml for 24 h) resulted in no significant cell cycle alterations (data not shown). To examine whether MB cells are capable of transiting through the cell cycle in the presence of NBT-272, cultures were synchronized at the G1/S boundary by using hydroxyurea treatment. The fractions of MB cells in G1, S, and G2/M at different times (0, 8, 16, and 24 h after release) were analyzed (Figure 6). After the hydroxyurea block most of the cells were found to accumulate in the G1 phase (Figure 6). In each case, the untreated control cells transited through S into G2/M phase by 8–16 h (DAOY by 8 h, D341 by 16 h). In contrast, flow cytometric analysis of synchronized MB cells treated with 0.4 μg/ml NBT-272 demonstrated arrest in G1 or S.

## Discussion

The present study shows for the first time that NBT-272 has anti-proliferative effects on human MB cell lines at nanomolar concentrations. DAOY cells with low c-MYC expression were slightly less sensitive to NBT-272 than D425 and D341 MB cells that are c-MYC amplified and express high levels of c-MYC. However, DAOY cells engineered to express increased levels of c-MYC showed only slightly enhanced sensitivity to NBT-272 when compared to parental DAOY cells. This indicates that the expression of c-MYC is not the only factor regulating the cellular sensitivity towards NBT-272. In an earlier study using leukemia and lymphoma cells, tumor cells expressing wild-type p53 and high c-MYC levels were most sensitive to bruceantin [22,25]. The results of the current study are in accordance with these observations. The slightly more sensitive MB cell lines D341 and D425 express wild-type p53 and high c-MYC levels, whereas DAOY cells harbor mutant p53 [40].

Our results also demonstrate that NBT-272 induces apoptosis in MB cells at submicromolar concentrations. The induction of apoptosis by NBT-272 was more prominent in MB cells expressing low c-MYC levels (DAOY wt, DAOY V11), giving no indication that c-MYC down-regulation is related to the susceptibility of MB cells to NBT-272 induced apoptosis. D341 and D425 human MB cells do not express caspase-8 [37] and DAOY cells harbor mutant p53 [40]. Neither caspase-8, nor p53 appear to be essential for NBT-272 induced apoptosis. Induction of apoptosis by quassinoids has been reported before [25,26,41]. Rosati et al. [40] have demonstrated that quassinoids can induce mitochondrial depolarization and caspase-3 activation. The results of the current study are in accordance with these findings. However, further studies are needed to elucidate the exact mechanisms of NBT-272-induced apoptosis in MB cells.

NBT-272 treatment of synchronized MB cells resulted in a block in cell cycle progression in MB cells, which may explain its anti-proliferative effects. The data presented in this report support the existence of quassinoid mediated effects on the cell cycle regulation. Bruceantin treatment of leukemia and lymphoma cells induced a G1 arrest in most cell lines tested [22,25]. It has been postulated that quassinoids inhibit c-MYC protein expression [25,26], which is confirmed in the present study. The ability of c-MYC to activate and repress target genes involved in cell-cycle progression has been investigated intensively [42-44]. Down-regulation of c-MYC expression by antisense prevented S-phase entry [45,46], indicating the important role of c-MYC in cell cycle progression. It has been suggested that quassinoids inhibit the purine synthesis pathway and inhibit DNA/RNA synthesis [47], and that inhibition of nucleotide synthesis may also contribute to the prolonged G1/S arrest. The exact mechanism by which NBT-272 inhibits cell cycle progression remains to be investigated.

The anti-proliferative effect of NBT-272 on MB cells was associated with c-MYC protein down-regulation and reduced c-MYC activity. The effect on c-MYC expression occurred at the post-transcriptional level, since the drug did not significantly affect c-MYC mRNA levels in MB cell lines. In MB, c-MYC gene amplification, aberrant signal transduction of the wingless (WNT) signalling pathway [48], or constitutive activation of STAT3 [49] are known mechanisms for c-MYC deregulation. In order to elucidate whether NBT-272 could influence these pathways, STAT3 activity was measured in cell lysates of cells treated with NBT-272. Our analysis revealed relatively low STAT3 activity in all MB cells and minor changes after NBT-272 treatment (data not shown). The NBT-272-mediated effect on c-MYC levels appears to be independent of STAT3 activity.

## Conclusion

In human MB cells, NBT-272 treatment inhibits cellular proliferation at nanomolar concentrations, induces apoptosis, and down-regulates the expression of the oncogene c-MYC. Thus, NBT-272 represents a novel candidate drug to inhibit proliferation of human MB cells *in vivo*.

## Competing interests

Dr. Lawrence Helson is an employee of TAPESTRY Pharmaceuticals. TAPESTRY Pharmaceuticals is the manufacturer of this compound and provided the NBT-272 employed in the study.

## Authors' contributions

AOVB performed most experimental work including data analysis and he drafted the manuscript. TS participated in the study design and supervised the experimental work. JR carried out most of the RT-PCR analysis. DS and CGE stably transfected the DAOY cell lines and participated in study design and data analysis. LH provided the drug and participated in the study design. MAG and AA lead the study and finalized the manuscript. All authors read and approved the final manuscript.

## Pre-publication history

The pre-publication history for this paper can be accessed here:
